# Reconfigurable ScAlN Piezoelectric Micromachined Ultrasonic Transducer Arrays for Range Finding

**DOI:** 10.3390/mi16020145

**Published:** 2025-01-26

**Authors:** Wenling Shang, Danrui Wang, Bin Miao, Shutao Yao, Guifeng Ta, Haojie Liu, Jinyan Tao, Xiaonan Liu, Xiangyong Zhao, Jiadong Li

**Affiliations:** 1 School of Nano-Tech and Nano-Bionics, University of Science and Technology of China, Hefei 230026, China; wlshang2021@sinano.ac.cn (W.S.); bmiao2013@sinano.ac.cn (B.M.); 2 Key Laboratory of Multifunctional Nanomaterials and Smart Systems, Suzhou Institute of Nano-Tech and Nano-Bionics, Chinese Academy of Sciences, Suzhou 215123, China; 3 School of Electrical and Mechanical Engineering, Changchun University of Science and Technology, Changchun 130022, China; 4 Key Laboratory of Optoelectronic Material and Device, Department of Physics, Shanghai Normal University, Shanghai 200234, China

**Keywords:** piezoelectric micromachined ultrasonic transducers (PMUTs), array, range finding, scandium-doped AlN (ScAlN)

## Abstract

Due to their compact sizes, low power consumption levels, and convenient integration capabilities, piezoelectric micromachined ultrasonic transducers (PMUTs) have gained significant attention for enabling environmental sensing functionalities. However, the frequency inconsistency of the PMUT arrays often leads to directional errors with the ultrasonic beams. Herein, we propose a reconfigurable PMUT array based on a Sc_0.2_Al_0.8_N piezoelectric thin film for in-air ranging. Each element of the reconfigurable PMUT array possesses the ability to be independently replaced, enabling matching of the required frequency characteristics, which enhances the reusability of the device. The experimental results show that the frequency uniformity of the 2 × 2 PMUT array reaches 0.38% and the half-power beam width (θ_−3dB_) of the array measured at 20 cm is 60°. At a resonance of 69.7 kHz, the sound pressure output reaches 7.4 Pa (sound pressure level of 108.2 dB) at 19 mm, with a reception sensitivity of approximately 11.6 mV/Pa. Ultimately, the maximum sensing distance of the array is 7.9 m, and it extends to 14.1 m with a horn, with a signal-to-noise ratio (SNR) of 19.5 dB. This research significantly expands the ranging capability of PMUTs and showcases their great potential in environmental perception applications.

## 1. Introduction

Airborne ultrasonic range-finders based on the principle of time-of-flight (ToF) have been widely utilized in sensing technology, such as three-dimensional (3D) object detection and tracking [1], automotive parking assistance [2], obstacle avoidance [3], and level measurement [4]. Compared to bulk piezoelectric ultrasonic transducers, piezoelectric micromachined ultrasonic transducers (PMUTs) based on microelectromechanical system (MEMS) technology have the characteristics of compact size, low power consumption, high integration with complementary metal oxide semiconductor (CMOS) technology, and good acoustic impedance matching [5].

Currently, the piezoelectric materials for PMUTs include polyvinylidene fluoride (PVDF), thin-film Pb(Zr, Ti)O_3_ (PZT), NaBiTiO_3_-BaTiO_3_ (NBBT), lithium niobate (LN), and aluminum nitride (AlN) [6,7,8,9,10]. In recent years, AlN PMUTs have attracted considerable attention in the field of ultrasonic range finding due to its high sensitivity, nontoxicity, and compatibility with CMOS manufacturing processes [11,12]. In 2009, Shelton et al. successfully applied the AlN PMUT to distance measurement for the first time [13]. By 2023, the ranging capability of the AlN PMUTs have gradually increased from 0.45 m to 9 m [14,15,16,17]. To further improve the performance of AlN thin films, doping Sc in AlN enhances the piezoelectric coefficient while maintaining a nearly constant dielectric constant [18,19]. Therefore, scandium-doped AlN (ScAlN) thin films can effectively fulfill the demanding requirements of both the transmission and reception performance of individual devices [20]. Ji et al. amplified the acoustic output and reception sensitivity by designing a Sc_0.2_Al_0.8_N PMUT with a bimorph dual-electrode structure [21]. Zhang et al. proposed a PMUT with a special dual-ring structure based on Sc_0.25_Al_0.75_N, achieving a sensing distance of 6 m for range finding at 91 kHz [22]. Li et al. increased the average output sound pressure by 17% by using the stepped-tube (expanded tube) backside cavity Sc_0.2_Al_0.8_N PMUT [23]. Yao et al. used a single Sc_0.2_Al_0.8_N PMUT based on flexurally suspended membrane equipped with a horn to achieve a record-breaking distance sensing range of 11.2 m [24]. Employing both the back cavity and the horn of the PMUT simultaneously can increase the transmission performance to a certain extent.

Compared to a single ScAlN PMUT, arrays are often used as a powerful method to achieve high sound pressure output for level measurements and unmanned aerial vehicle ground measurements. The outstanding research is a PMUT array composed of 14 designed Sc_0.2_Al_0.8_N PMUT elements reported by Yang et al., which can reach a distance of 6.8 m at 66 kHz [25]. However, the frequencies of the elements in traditional integrated PMUT arrays are not uniform, which prevents the arrays from achieving maximum performance. The damaged elements in an array cannot be replaced, indicating that the array lacks flexibility. The structural optimization of PMUTs can be used to solve the problem of the inherent frequency uniformity of arrays, such as dummy cavity structures and cantilever cluster structures [26,27]. However, compared with the traditional structure [28], although the structural optimization of PMUT can improve frequency uniformity, its transmission sensitivity has no advantages. Therefore, it is challenging for PMUT devices to have both high frequency uniformity and great sound pressure emissions. Meanwhile, researchers have improved the accuracy of ultrasonic detection by improving the acoustic directivity of PMUT arrays. For example, Yang et al. added a horn to a PMUT array to concentrate the acoustic energy and increase the sound pressure of the transducer in the direct front [25]. Li et al. used a stepped-tube backside cavity to enhance the directivity of the acoustic field [23]. However, due to the limitations of MEMS fabrication processes, few reports have been made about the acoustic directivity of PMUT array devices with a pitch of half a wavelength.

In this paper, we propose a reconfigurable PMUT array (R-PMUT) for in-air long- range finding. The array is reconstructed from high-performance Sc_0.2_Al_0.8_N PMUT elements, exhibiting the characteristics of high frequency uniformity and good directivity. The reconfigurable feature allows elements to be updated and replaced, increasing the reusability and flexibility of the device. The device performance characterization and ranging results indicate that the Sc_0.2_Al_0.8_N reconfigurable PMUT array significantly improves the sound pressure output and ranging capability.

## 2. Design and Fabrication

### 2.1. Design of the PMUT

**Figure 1:**
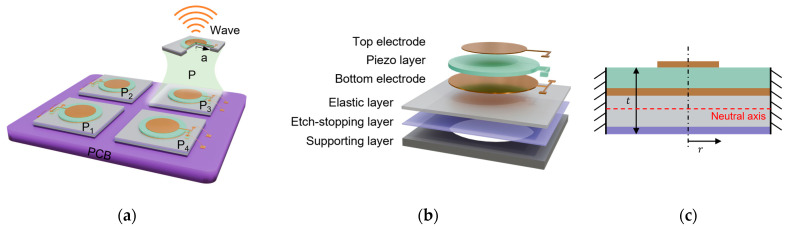
(**a**) Diagram of a reconfigurable PMUT array (R-PMUT, P_1_–P_4_ are four elements of the R-PMUT.); (**b**) exploded view of a clamped PMUT; (**c**) schematic diagram of the boundary structure.

Figure 1a provides a schematic diagram of the R-PMUT, where a single PMUT consists of a supporting layer, an etch-stopping layer, an elastic layer, a bottom electrode, a piezoelectric layer, and a top electrode from bottom to top, as shown in Figure 1b. Applying voltage between the top electrode and the bottom electrode generates radial force in the piezoelectric layer. The vibrating diaphragm operates in the flexural vibration mode of d_31_ and generates pressure longitudinal waves. Due to the significant difference in the acoustic impedance between air and general objects, such as solids and liquids, most ultrasonic waves are reflected. The echo ultrasonic signal is reflected by the target object and reaches the surface of the sensor. The piezoelectric thin film converts pressure into electrical signals due to the piezoelectric effect. Then, the system measures the distance of the object based on the ToF principle to sense the surrounding environment.

The ultrasound frequency mainly determines the propagation distance. The ultrasound absorption coefficient is directly proportional to the square of the frequency, indicating that low frequencies result in slow attenuation and great propagation distances [29]. If the frequency is overly low, the wavelength of the sound wave is overly long, resulting in a decrease in the measurement accuracy [30]. The maximum detection range can be estimated by the acoustic propagation loss [31]; for example, with a reference distance of 20 cm, a PMUT operating at a frequency of 70 kHz loses 60 dB at 4 m.

The performance of the Sc_0.2_Al_0.8_N PMUT element can be analyzed by using a circuit model. For a circular membrane functioning as a clamped plate, the approximate first-order mode shape, $w\left(\bar{r}\right)$, is as follows [32]:

(1) $$w\left(\bar{r}\right)=w\left(0\right){\left(1-{\bar{r}}^{2}\right)}^{2},$$

where $\bar{r}=r/a$, *a* is the radius of the membrane, and *r* is the distance from the center of the membrane. The mechanical stiffness *k_m_* and electromechanical coupling coefficient *η* are derived from the piezoelectric energy and elastic strain energy generated by the work performed by the piezoelectric force in the film [33]:

(2) $$k_{m}=\frac{64\pi D}{3a^{2}},$$

(3) $$\eta =-\pi e_{31,f}\bar{z_{p}},$$

where *D* is the flexural rigidity; *e*_31,*f*_ = −1.6 C/m^2^ is the piezoelectric coefficient of Sc_0.2_Al_0.8_N [21]; and $\bar{z_{p}}$ is the distance from the middle of the piezoelectric layer to the neutral axis.

The resonant frequency *f*_0_ of the first-order mode is calculated as follows:

(4) $$f_{0}=\frac{10.327}{a^{2}}\sqrt{\frac{D}{\mu }}\propto \frac{t}{a^{2}},$$

where *µ* is the area mass density and *t* is the thickness of the membrane. At a fixed frequency, the radius is maximized, the thickness is minimized, and the film thickness is adjusted to achieve zero stress, which is helpful for enhancing the vibration displacement of the film [14]. The amplitude of the vibration at the center of the film due to an input voltage *V_in_* can be approximated as follows:

(5) $$w(0)\approx \frac{\eta V_{in}}{k_{m}}\propto \frac{e_{31,f}\bar{z_{p}}a^{2}}{D}.$$

**Figure 2:**
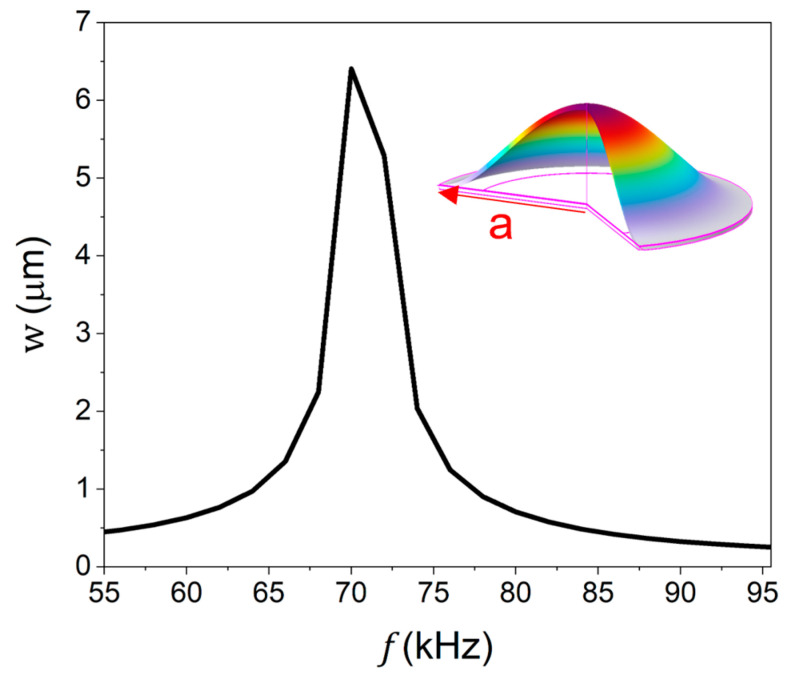
Frequency response of the PMUT: center displacement *w* at different frequencies (inset: first-order vibration mode, and *a* is the radius of the membrane).

With the same structural size, the vibration amplitude of the Sc_0.2_Al_0.8_N PMUT is twice that of the AlN PMUT. COMSOL Multiphysics 6.0 simulation is used to simulate the frequency response of the central vibration displacement of the PMUT element (top electrode Au/piezo layer ScAlN/bottom electrode Mo/elastic layer Si: 0.2 µm/1 µm/0.2 µm/5 µm, a: 780 µm), and the maximum displacement reaches 6.4 µm, as shown in Figure 2.

The far-field sound pressure *p* on the axis of the PMUT array composed of *N* elements is as follows [29]:

(6) $$p\left(x\right)\approx j\omega \frac{N\rho_{0}u_{0}a^{2}}{6x}{\mathrm{e}}^{j\left(\omega t-kx\right)}\propto \frac{Nw\left(0\right)}{x},$$

where *x* is the distance from the sound source, *j* is the imaginary part, *ρ*_0_ is the density of air, *u*_0_ is the vibration velocity of the membrane, *k* is the wave number defined as $k=\frac{\omega }{c_{0}}$, *ω* is the angular frequency, and *c*_0_ is the propagation speed of sound waves in the air. Under the same structure, the thickness of the thin film is approximately the same. Therefore, when the frequency of the array elements is uniform, the membrane radius is approximately equal, and the vibration amplitude is almost the same, enhancing the sound pressure of the array by N times compared to an element. In practice, the typical operational bandwidth of a PMUT in air is approximately 1 kHz, rendering it a device with narrowband characteristics [27]. Residual stress and process deviation often reduce the frequency uniformity of the device, resulting in unsatisfactory output performance of the array. Therefore, frequency uniformity is essential for maximizing the performance of an array when it is used to enhance the output sound pressure. In this paper, the reconfigurable device shown in Figure 1a is constructed by selecting elements with the same frequency, using a 2 × 2 small-scale array for illustration, which can increase the sound pressure four-fold.

To avoid grating lobes and sidelobes, the array pitch is slightly less than $\frac{\lambda }{2}$ [29]. When close elements are combined, they affect each other, and the sound intensity *I* becomes N times that when the elements are dispersed:

(7) $$I=\frac{N^{2}p^{2}}{2\rho_{0}c_{0}}.$$

**Figure 3:**
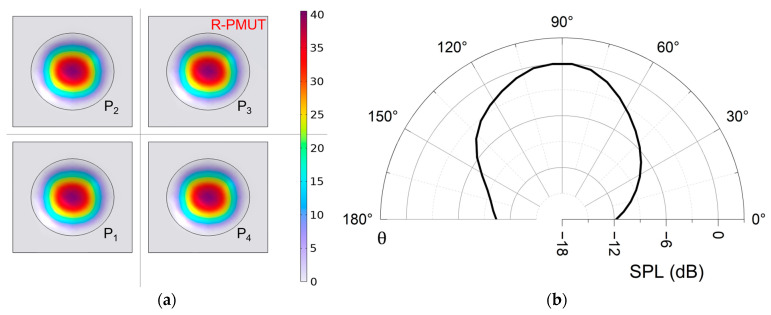
(**a**) First-order modal simulation of a R-PMUT, where the color of the legend represents the magnitude of the vibration amplitude, and P_1_–P_4_ are four elements of the R-PMUT; (**b**) simulation of directivity normalized of far-field sound pressure level (SPL) for the R-PMUT.

In addition, the intensity and directivity of sound waves can be affected by unpredictable variations in the vibration phase and amplitude caused by crosstalk between elements in arrays. The primary cause of crosstalk is acoustic coupling [34]; therefore, to mitigate crosstalk between elements in an array, it is necessary to implement element separation. Under the premise of a pitch of $\frac{\lambda }{2}$, the spacing between the elements forms grooves, which suppress crosstalk by reducing the acoustic coupling between the elements by increasing the air domain. The first-order modal simulation of a R-PMUT is shown in Figure 3a, indicating high consistency in vibration amplitude. The sound directivity of the R-PMUT at 20 cm is shown in Figure 3b, with a −3 dB beamwidth of 61.7°.

### 2.2. Fabrication of the PMUT

**Figure 4:**
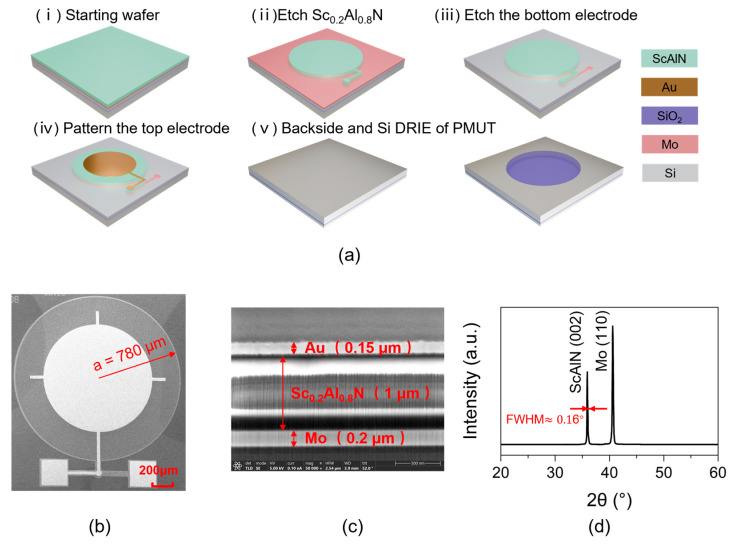
(**a**) Fabrication process flow of the PMUT; (**b**) SEM image of the PMUT element; (**c**) SEM image of each layer at the central position of the PMUT; (**d**) XRD pattern of the ScAlN thin film.

Figure 4a(i–v) shows a flowchart of the fabrication process for the latest PMUTs we have developed, as mentioned in a previous work [22]. The designed PMUT is fabricated from the piezoelectric film Sc_0.2_Al_0.8_N, which is grown on an SOI wafer (provided by Shanghai Normal University), as shown in Figure 4a(i). In addition, ii and iii involve sequentially etching the ScAlN layer using ion beam etching and the Mo layer using plasma etching, respectively, from top to bottom, to form a patterned thin film. The whole surface is sputtered with gold, and the top electrode is patterned by the lift-off process (Figure 4a(iv)). Figure 4a(v) shows the process of etching the backside of the device using deep reactive ion etching (DRIE), until it reaches the SiO_2_ etch-stopping layer. Here, the radius of the back cavity defines the radius of the PMUT film. Figure 4b displays an SEM image of the PMUT element fabricated with a 780 µm radius. The thickness and quality of each layer of the PMUT are shown in Figure 4c. The bright and dark light in the ScAlN layer arise due to the accumulation of charges. XRD analysis reveals the presence of a diffraction peak at approximately 36°, corresponding to the (002) orientation of the ScAIN thin film, as depicted in Figure 4d. The half-height width for this peak is approximately 0.16°. Additionally, a diffraction peak attributed to the (110) orientation of the Mo film is observed near 40°. The sharp diffraction peak of ScAlN indicates good crystal orientation and high crystal quality.

**Figure 5:**
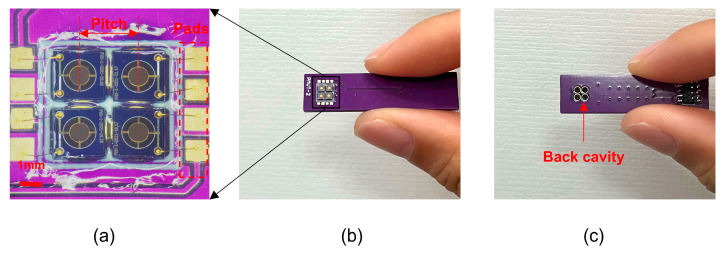
(**a**) Optical image of a 2 × 2 R-PMUT; (**b**) front of the device; (**c**) back of the device.

The PMUT element has a membrane radius of 780 μm, occupying an area measuring 2 mm × 2 mm. The larger the aperture of the array is, the stronger the sound pressure, and the better the directivity. Considering the complexity of both the spatial arrangement of the pads and the circuit, the 2 × 2 arrangement is chosen. The pitch of the elements is 2.45 mm. The PMUT elements are tested using an impedance analyzer, and PMUT elements with approximately equal frequency and impedance values are selected to assemble a 2 × 2 PMUT array device. Then, the PMUT elements are glued onto the printed circuit board (PCB, printed by Shenzhen JLC Electronics Company Limited, Shenzhen, China) with epoxy adhesive (provided by Dongguanshi TEGU New Materials Company Limited, Dongguan, China), as shown in Figure 5a. In addition, the PMUT array device can be reused by replacing the damaged PMUT elements with good PMUT elements of the same frequency. Therefore, using this reconfigurable implementation method, which mainly includes frequency selection and recombination of elements, the frequency uniformity and reusability of the sensor can be significantly enhanced. The front and back of the device are shown in Figure 5b,c, respectively. The round-trip sensitivity can be improved by using the back cavity of the device to transmit and receive sound waves [28]. Therefore, in this experiment, the back cavity of the PMUT is used to transmit and receive sound waves, and the back cavity is designed to have a radius of “a” and a length equal to the sum of the thickness of the silicon support layer (280 µm) and the thickness of the PCB (1 mm), which is approximately a quarter of the wavelength [35].

## 3. Ultrasonic Detection System

**Figure 6:**
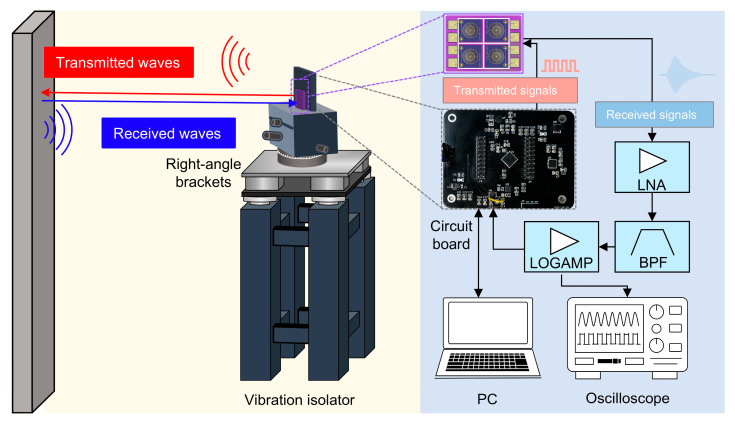
System diagram and measurement setup for ranging.

The framework of the system assembly is shown in Figure 6. Pulse-echo measurements are adopted. Using the program configured by the computer and the serial peripheral interface (SPI) communication protocol, the burst signals of a specific frequency determined by the internal clock of the chip are applied to the electrode of the device. The electrical signal is converted into a mechanical wave signal by a piezoelectric thin film. The acoustic wave is emitted from the surface of the membrane and travels through the propagation medium, such as air, until it encounters the detected target, where it subsequently turns into a reflected acoustic wave back to the surface of the vibrating diaphragm of the PMUT, which causes mechanical vibration which is converted into electrical signals [35]. Subsequently, the electrical signal is captured by the circuit.

**Figure 7:**
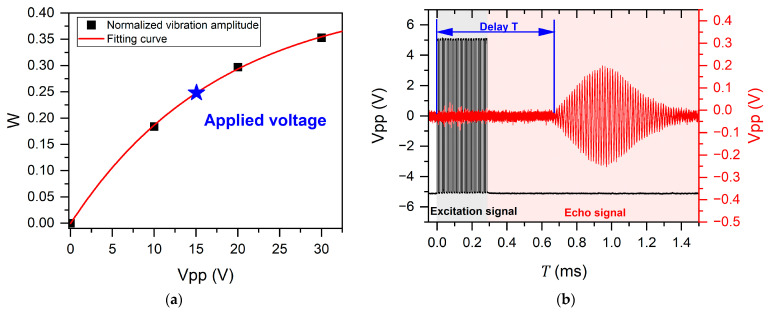
(**a**) Normalized vibration amplitude *W* of devices driven by different voltages *Vpp*; (**b**) variation of the excitation pulse signal and the echo signal with time *T*.

As shown in Figure 7a, the vibration amplitude of the piezoelectric thin film increases as the voltage increases, albeit with a gradual deceleration rate. Moreover, within the constraints of low power consumption, there is a limitation on the excitation voltage amplitude. Additionally, the propagation of ultrasonic waves in air exhibits exponential attenuation, resulting in a weak echo amplitude [36]. Therefore, the PMUT array is utilized as both a transmitter and receiver to mitigate the frequency mismatch between the transmitted and received signals. In addition, the circuit that captures the signal must consist of a low-noise linear amplifier, a bandpass filter, and a logarithmic gain amplifier to capture weak echo signals and provide an excellent input dynamic range over the entire range of reflected waves [37]. In this manner, a demodulated analog output representing the received echo, the zero crossing of the input signal, and a simple threshold crossing indicator all enable presence detection and considerable signal-to-noise ratio (SNR). Then, the obtained analog signals are converted into digital signals by analog-to-digital converters (ADCs) and transferred to microprogrammed control units (MCUs) or personal computers (PCs). The amplitude of signals and pertinent time information can be directly observed through an oscilloscope. The total test system is mounted on a vibration isolator to minimize interference from ambient vibration, which effectively enhances the SNR of the signal.

Figure 7b shows the voltage excitation signal applied to the PMUT and the received echo signal. The rising edge signal of the echo signal is considered to be the starting point of the travel time [38]. We obtain the maximum value of the voltage amplitude of the echo signal via the peak-detection algorithm and the associated time information. In addition, we calculate the measured distance through the ToF principle. The whole system is designed to ensure the smooth operation of the experiment with testability and portability.

## 4. Results and Discussion

### 4.1. PMUT Performance

**Figure 8:**
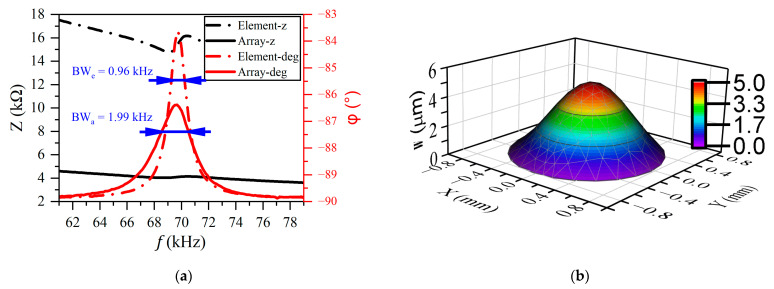
(**a**) Measured electrical impedance values *Z* and phase *φ* of a PMUT element and a PMUT array; (**b**) first-order vibration mode of the PMUT element.

The resonant frequency of the device was evaluated by electrical impedance measurements, which were measured by a precision impedance analyzer (MICRO-TEST 6632, Taiwan, China). The resonant frequencies of the PMUT element (solid red line) and the array (dashed red line) were approximately the same at 69.7 kHz, as shown in Figure 8a. The bandwidth is defined as the width of the frequency range with a maximum phase drop of 70.7% [39]; thus, the bandwidths of the element PMUT and R-PMUT were 0.96 kHz and 1.99 kHz, respectively. The frequency uniformity of five R-PMUT arrays was 0.16%, 0.41%, 0.80%, 0.12%, and 0.43%. The reliability of the reconfigurable array implementation method is demonstrated, and the frequency uniformity is as expected.

A decrease in the stiffness of Sc-doped AlN films leads to an increase in the vibration amplitude of the piezoelectric films, thus increasing the output of sound pressure [40,41]. Under continuous square wave excitation with a voltage of 10 V and a frequency of 69.7 kHz, the first-order vibration mode of the R-PMUT thin film was measured, as shown in Figure 8b. Under continuous square-wave excitation with a voltage of 10 *Vpp* and a frequency of 69.7 kHz, the mechanical vibration morphology and amplitude of R-PMUT devices were measured with a microscopic laser vibrometer (MSA-600-XU, Polytec, Waldbronn, Germany), and the maximum vibration displacement was 5.3 µm, as shown in Figure 8b.

At a drive voltage of 15 *Vpp*, the sound pressure output of the element PMUT and the R-PMUT were 2.2 Pa and 7.4 Pa, respectively, measured using a calibrated reference microphone (Gras 46BF-1, Holte, Denmark) at a distance of 19 mm from the device. The reception sensitivity of the element PMUT and R-PMUT were found to be 8.8 mV/Pa and 11.6 mV/Pa, respectively, by using the corrected PMUT to emit ultrasonic waves.

**Figure 9:**
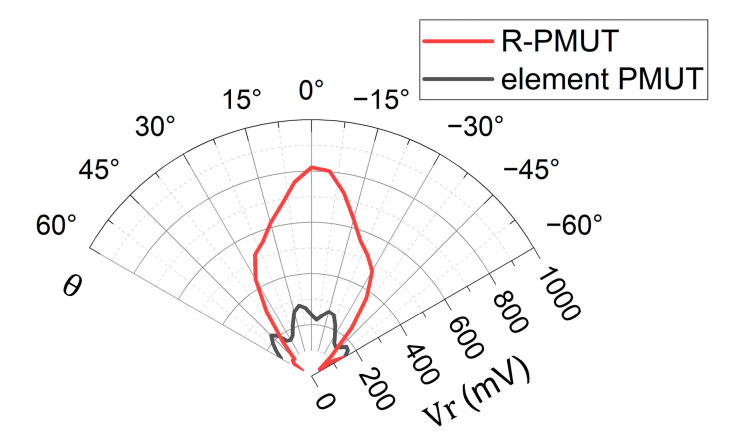
Measurement of the directivity of far-field sound for the element PMUT and R-PMUT.

To characterize the beam directivity of the PMUT device, the measured PMUT array was fixed at the origin of a three-dimensional space, took the *z*-axis as the rotation axis, rotated 120° from the −*y*-axis to the +*y*-axis direction in the xy-plane, in steps by 5°, and emitted ultrasonic waves. A calibrated PMUT was fixed in the +x direction, received sound waves at a distance of 20 cm from the device, and converted the acoustic signal into an electrical signal, *Vr*. The results show that the half-power beam width (θ_−3dB_) of the R-PMUT was 60°, as shown in Figure 9. This is because the pitch of the elements of R-PMUT can be precisely set to half wavelength, preventing the dispersion of sound energy by the gate and side lobes, so that the sound energy is more concentrated forward.

### 4.2. Ultrasonic Ranging

**Figure 10:**
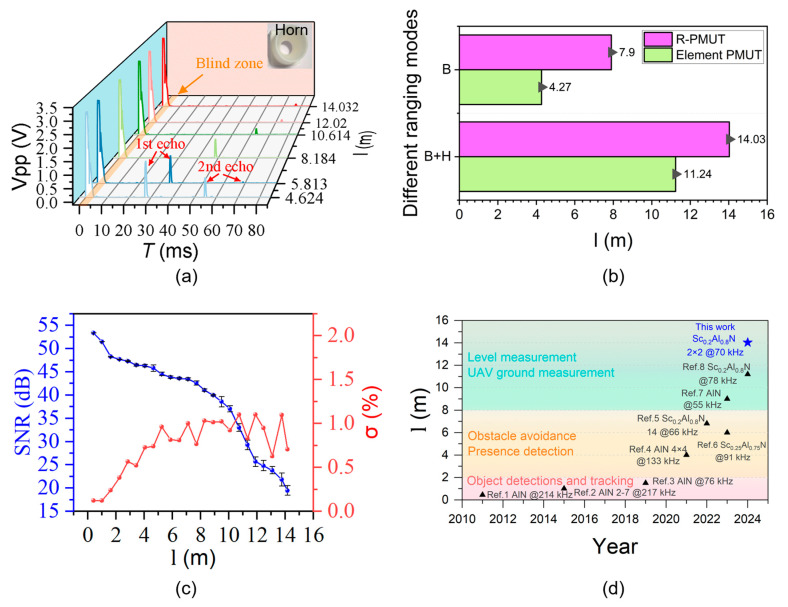
(**a**) Signal waveform of the R-PMUT pulse-echo signal; (**b**) farthest sensing distances *l* of the R-PMUT and element PMUT with a resonant frequency of 69 kHz (B: back cavity, H: horn); (**c**) signal-to-noise ratio (SNR) vs. distance: at the threshold of 19.5 dB, the maximum range of the PMUT array is 14 m (temperature: 15 °C, relative humidity: 37%); (**d**) comparison of the maximum sensing distances (Data are from Ref. 1 ([14]), Ref. 2 ([28]), Ref. 3 ([15]), Ref. 4 ([17]), Ref. 5 ([25]), Ref. 6 ([22]), Ref. 7 ([16]), Ref. 8 ([24])).

Finally, the ranging performance of the PMUT device was evaluated. Ultrasonic ranging is based on the ToF principle, which involves emitting ultrasonic waves into the air and receiving the echo signal after a delay T and the distance $d=\frac{cT}{2}$. To facilitate the test, a wall was chosen as a reflector. In addition, the utilization of acoustic horns augmented the energy concentration along the propagation axis, consequently enhancing the sound radiation power and improving the ranging capabilities [16,22]. Here, we chose horns (H) which had a throat radius of 0.4 cm, a field of view angle of 30°, and heights of 2.0 cm. The system in Figure 6 was used for ultrasonic ranging, and the results are displayed. The R-PMUT with a horn in the back cavity (B) was driven by 30 cycles of 15 *Vpp*, 69 kHz pulsed square waves with 50 ms intervals. The envelope of transmitting ultrasonic signal and receiving echo signal was displayed on the oscilloscope, as shown in the 3D waterfall diagram in Figure 10a. As shown in the orange area, the blind area reached 71.9 cm. Due to the enhancement of the signal caused by the horn, the second echo signals could be clearly observed at a distance of 5.8 m and closer. This observation was apparent because the volume of the detection equipment was overly large. Thus, the first echo continued to reflect, which was regarded as transmitting ultrasound and as a second distance detection. This time was exactly twice that of the first echo. The maximum range of the device was determined by the SNR [33]. For this experiment, the maximum distance that the experimental environment could reach was 14.5 m at an SNR of 19.5 dB, as indicated by the red line in Figure 10a.

Figure 10b shows the maximum ranging distance of an element PMUT and R-PMUT with an SNR of 19.5 dB. The ranging effect of the array is obviously stronger than that of the element. Using the back cavity and the horn of the device can significantly improve the ranging performance. In addition, to evaluate the accuracies of the measurement points and detection distances, we added a standard laser distance meter (LM100-A, UNI-T Instruments, Shenzhen, China) to analyze the ranging error. Figure 10c shows that the relative error *σ* of the ultrasonic distance measurement increases with increasing distance. When measuring long distances, the measurement error increases due to the decrease in the SNR. Figure 10d presents a comparison of other papers featuring PMUT ranging studies with ours, indicating that our distance measurement is more advanced among the state-of-the-art methods.

## 5. Conclusions

In summary, a reconfigurable PMUT array capable of performing long-range ranging in air is reported. The PMUT elements achieve a maximum vibration amplitude of 5.3 µm by doping 20% Sc in the AlN film, and the reconfigurable PMUT array has excellent transmission sound pressure performance. The reconfigurable PMUT arrays prepared by this method have relatively high frequency uniformity and good directivity. Therefore, these arrays achieve a distance sensing of 14.03 m at an SNR of 19.5 dB at a resonant frequency of 69 kHz within an area of 4 mm by 4 mm. In addition, the elements of the reconfigurable PMUT array are replaceable, increasing the reusability of the devices. Overall, this PMUT array demonstrates the ability for long-distance detection in the air, making it possible to integrate it into microintelligent devices for additional distance sensing applications.

## Data Availability

The raw data supporting the conclusions of this article will be made available by the authors on request.
